# Measurement Matters: MRI Analysis of Differing Anatomic Measurement Techniques for Velar Length and the Velopharyngeal Needs Ratio

**DOI:** 10.1097/GOX.0000000000005617

**Published:** 2024-02-19

**Authors:** Kazlin N. Mason, Bailey Hanson, Jonathan S. Black

**Affiliations:** From the * Department of Human Services, University of Virginia, Charlottesville, Va.; † Division of Plastic Surgery, University of Virginia Health System, Charlottesville, Va.

## Abstract

**Background:**

Advances in imaging tools provide opportunities to enhance how velopharyngeal (VP) variables are quantified to facilitate surgical decisions. The purpose of this study was to use magnetic resonance imaging (MRI) to determine if quantitative differences were present between measures of linear and curvilinear velar length, and subsequently, the VP needs ratio.

**Methods:**

Data were prospectively collected from patients presenting with repaired cleft palate and/or congenital palatal insufficiency with or without VPI at a single center tertiary children’s hospital. Quantitative measures of the velopharynx using a novel nonsedated MRI protocol were obtained. Paired samples *t* tests were conducted to assess if differences were present between the VP needs ratio and measurements of linear and curvilinear velar length at rest and during sustained phonation. Intraclass correlation coefficients were calculated to assess intra/inter-rater reliability.

**Results:**

Significant differences were present between measurements of linear and curvilinear velar length at rest (*P* ≤ 0.001) and during sustained phonation (*P* ≤ 0.001). Significant differences were also present in the VP needs ratio (*P* ≤ 0.001). Curvilinear velar length at rest and during sustained phonation was longer than that of linear velar length at rest and during sustained phonation. No significant differences were observed between measures of effective velar length (*P* = 0.393).

**Conclusions:**

Measurement differences influence the VP needs ratio. This may have implications for comparisons to previously reported normative reference values and for those who are anatomically at risk for VPI. MRI provides an enhanced imaging modality to assess normative benchmarks and the anatomic variables used to define VP anatomy for clinical decision-making.

**Takeaways**

**Question:** Incorporating anatomic data into the assessment process for children with velopharyngeal insufficiency is crucial for improving speech outcomes. How data are obtained and measured may influence clinical decisions that are made.

**Findings:** Differences are present between linear and curvilinear measures. Curvilinear measurements track the precise morphological variability of the soft palate. Data reported in this study lend preliminary support for consistent application of curvilinear measurements and have the potential to benefit future research and assessment models for predicting patient-specific outcomes.

**Meaning:** Curvilinear measurements from magnetic resonance imaging data provide greater accuracy for anatomic assessment. Application of this measurement technique will influence the calculation of the velopharyngeal needs ratio.

## INTRODUCTION

Anatomic measures for velopharyngeal (VP) variables have characteristically been derived from lateral radiographs. However, recent advances in imaging modalities and measurement tools have provided opportunities to enhance how key VP variables are visualized, measured, and reported. In the past decade, magnetic resonance imaging (MRI) studies have quantified VP variables in the cleft and noncleft populations and tracked change associated with growth and differing cleft types.^1–9^ Analyses related to outcomes after surgical procedures have also been reported.^5,10,11^ A key metric that has been consistently reported across studies is that of velar length.

The velum plays a central role in the complex process necessary in achieving normal speech and balanced resonance. Adequate velar length, in addition to the coordination of multiple anatomic structures, facilitates successful VP closure. The length of the soft palate has been reported to vary by age, race, and sex and is further influenced by palatal anomalies, such as cleft palate, and other craniofacial conditions.^12–16^ Thus, velar length has become a significant consideration for assessing VP function. The measure of effective velar length has also been reported to be a clinically relevant measurement.^4,17–21^

Seminal work by Subtelny^13^ highlighted that measures of pharyngeal depth and velar length facilitate assessment of the anterior to posterior dimension of the soft palate. The combination of these measures is frequently used to determine if the soft palate structure is sufficient to enable a patient to achieve VP closure. This defines the VP needs ratio, which is calculated by the measurement of pharyngeal depth in the numerator and soft palate length in the denominator^12–14,22–26^or, in more recent work, as the ratio of soft palate length divided by pharyngeal depth.^6,10,17,20,27–29^ Thus, the VP needs ratio has been supportive as an anatomic indicator for the ability to achieve VP closure during speech.^12,13,23,30–32^ Notably, within these seminal works, a measure of *linear* velar length is often described and measured as a straight line from the posterior maxillary point (PMP) or posterior nasal spine (PNS) to the uvular tip. This is common in clinical settings.^22,27,33–40^ Additionally, many prior studies, predominantly those that reference lateral cephalograms and computed tomography imaging modalities, use a linear measure of velar length as well.^13,22,27,35,37^

In contrast, Kuehn and Kahane^41^ documented a method for measuring the velum, resulting in a measurement that followed the anatomic curve of the soft palate. This measurement of velar length is defined as *curvilinear* velar length and measured as a curved line from the anterior to posterior segment of the velum along the midline region between the oral and nasal velar surfaces. Subsequent studies, often using MRI, frequently report measures of curvilinear velar length.^1–3,15,17–19,21,42–47^ Nasal surface measures of velar length during phonation have also been reported.^36,48,49^ However, variability, both in the definitions provided and measurement technique applied for velar length, is consistently observed across the literature. Despite differences in definition and execution of the measurement, many studies often refer only to the measure as “velar length” without providing specificity for use of a linear or curvilinear measure. It is unknown if measurements of velar length, effective velar length, and subsequently the VP needs ratio, are influenced by linear or curvilinear measurement techniques.

Given the need for accurate measurement to develop reference norms and facilitate clinical decision-making, consistency in definition and methodology is warranted. With advances in MRI, visualization software, and measurement tools, improved accuracy for measurement of velopharyngeal structures is readily obtained. The purpose of this study was to use MRI to determine if quantitative differences were present between linear and curvilinear measures of velar length, effective velar length, and the resulting VP needs ratio for children with repaired cleft palate and/or velopharyngeal insufficiency (VPI).

## METHODS

### Participants and Data Acquisition

In accordance with the institutional review board (HSR#200333), 25 patients with repaired cleft palate with or without lip involvement (CP ± L) and/or congenital palatal insufficiency with or without VPI were prospectively recruited from a tertiary-hospital-based craniofacial clinic. All participants were aged between four and 17 years (M = 9.74 ± 4.77 years). Participants were free of hearing and neuromuscular disorders.

Data were acquired using a high-resolution, T2-weighted, anatomic scans of the velopharyngeal region using turbo-spin echo sequences with variable flip angle. Participants were scanned in the supine position using a Siemens 3-Tesla Prisma scanner and a 64-channel head coil. Three-dimensional (3D) and two-dimensional velopharyngeal data were acquired at rest and during sustained phonation. The 3D anatomic whole-head scan was obtained with the velum in a relaxed and lowered position. Phonation data were achieved using productions of sustained /s/ and /i/. Two-dimensional images were acquired in the midsagittal, axial, and oblique coronal planes. Scanning sequences allowed for full acquisition of imaging data in less than 15 minutes per participant (approximately 4 minutes for 3D scans and 8–11 seconds for each phonation scan). Scanning parameters and data acquisition are consistent with those previously reported in the literature.^3,21,50^ Involvement of the speech-language pathologist was essential when obtaining and evaluating the velopharyngeal MRI data. Measurement definitions for all variables of interest are defined in Table 1.

**Table 1.** Velopharyngeal Variables of Interest

Measurement	Definition
Linear velar length	A straight line from the posterior maxillary point (PMP) or posterior nasal spine (PNS) to the uvular tip on the midsagittal image plane
Curvilinear velar length	A curvilinear line from the anterior to posterior segment of the velum along the midline region between the oral and nasal velar surfaces on the midsagittal image plane
Pharyngeal depth	Linear distance between the PNS or PMP and posterior pharyngeal wall (or adenoid) at the level of the palatal plane as seen on the midsagittal image plane
Linear effective velar length	A straight-line distance from the PNS/PMP to the middle of the levator veli palatini (LVP) muscle where it inserts into the body of the velum on the midsagittal image plane
Curvilinear effective velar length	A curvilinear distance from the PNS/PMP to the middle of the LVP muscle where it inserts into the body of the velum on the midsagittal image plane
Linear VP needs ratio	Ratio calculated by dividing the measurement of pharyngeal depth with the measurement of linear velar length
Curvilinear VP needs ratio	Ratio calculated by dividing the measurement of pharyngeal depth with the measurement of curvilinear velar length

### Image and Statistical Analyses

**Fig. 1.**
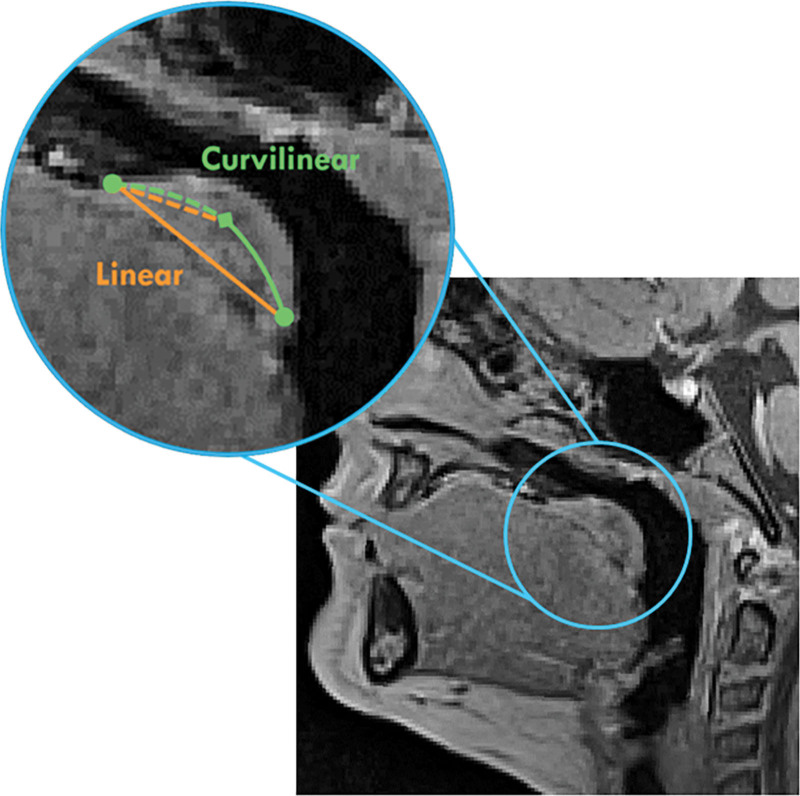
Comparison between linear (orange) and curvilinear (green) measures of velar length (solid lines) and effective velar length (dashed lines) on midsagittal MRI. The superior circular dot (green) indicates the location of the PMP, which is used as the starting point for measurements. The uvular tip is noted by the inferior circular dot, which notates the final landmark for measurements. The midline of the levator veli palatini muscle, as viewed on a midsagittal slice, is indicated by the diamond (green). This landmark can be used to obtain measurements of effective velar length as well as to track the course of the curvilinear velar length measurement.

MRI data were imported into 3D Slicer (Version 5.2) for visualization and quantification.^51,52^ Of the 25 participants, two were excluded from analyses due to increased motion during the scan, resulting in reduced visualization of the MRI data. Therefore, N = 23 scans were available for analyses. Images were randomized and measured by two raters with experience quantifying velopharyngeal anatomy. Two-way mixed consistency single-measures intraclass correlation coefficients (ICCs) were calculated for all linear and curvilinear measurements to assess inter- and intrarater reliability.^53,54^ Data were analyzed using R and RStudio.^55^ Paired samples *t* tests were conducted to assess if differences were present between the VP needs ratio and measurements of linear versus curvilinear velar length at rest and during sustained phonation. These measurements were made using the markups module in 3D Slicer. Figure 1 highlights the measures and landmarks for obtaining linear versus curvilinear measurements.

## RESULTS

Twenty-three patients completed adequate imaging data acquisition and were included in the data analyses. Reliability measures using ICCs demonstrated good to excellent inter- and intrarater reliability across all measurements (ICC κ range = 0.89–0.97). ICCs for inter-rater reliability of linear velar length and curvilinear velar length at rest were 0.94 and 0.91, respectively. ICCs for inter-rater reliability for linear velar length and curvilinear velar length during phonation were 0.92 and 0.89, respectively. Measurements of pharyngeal depth demonstrated excellent inter-rater reliability (κ = 0.97).

**Fig. 2.**
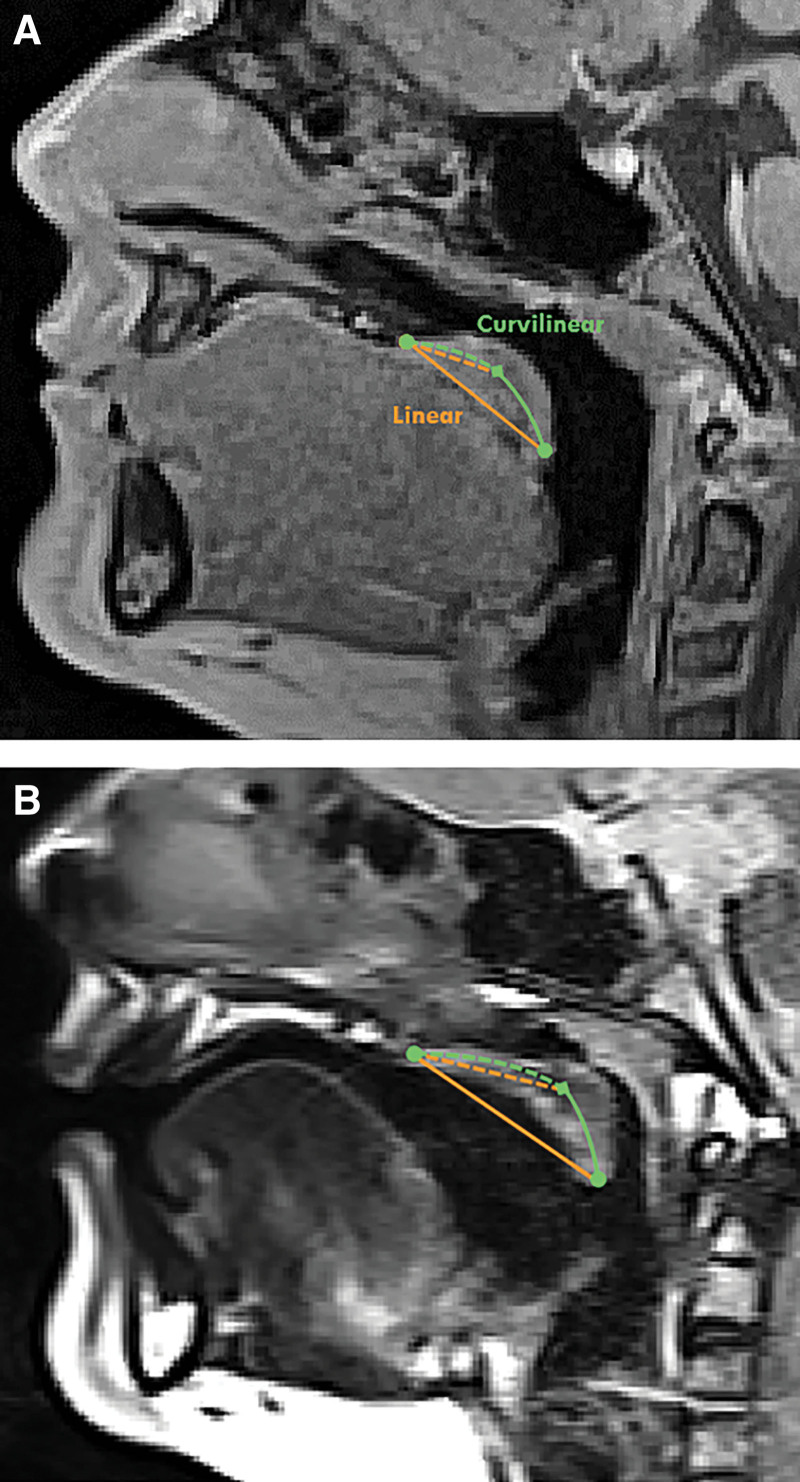
Demonstration of an individual with VPI comparing the soft palate at rest (A) and during phonation (B) with linear (orange) and curvilinear (green) measurements. Measurement of this structure is ideally captured using the curvilinear measurement of velar length.

Significant differences were present between measurements of linear velar length compared with curvilinear velar length at rest [t(22) = −4.101; *P* = <0.001] and during sustained phonation [t(22) = −3.953; *P* = <0.001]. Figure 2 illustrates the difference between linear versus curvilinear measurements at rest and during phonation. There was a strong positive correlation between measurements of linear velar length at rest and curvilinear velar length at rest [r(23) = 0.937; *P* < 0.001].

Significant differences were also present in the VP needs ratio between calculations using linear versus curvilinear measures [t(22) = 4.648; *P* = <0.001]. Additionally, across every measurement, curvilinear velar length at rest (M = 24.19; SD = 4.8mm) and during sustained phonation (M = 21.52; SD = 5.98mm) was found to be longer than that of linear velar length at rest (M = 22.75; SD 4.5 mm) and during sustained phonation (M = 20.31; SD = 5.08 mm). No significant differences were observed between linear and curvilinear measures of effective velar length [t(22) = 0.871; *P* = 0.393]. Results are reported in Table 2.

**Table 2.** Results

Variable of Interest	Mean (mm)	SD (mm)	T	DF	*P*
Curvilinear velar length (rest)	24.19	4.80	–4.101	22	<0.001*
Linear velar length (rest)	22.75	4.50			
Curvilinear velar length (phonation)	21.52	5.98	3.953	22	<0.001*
Linear velar length (phonation)	20.31	5.08			
Curvilinear effective velar length	11.57	3.82	0.871	22	0.393
Linear effective velar length	11.25	4.09			
Curvilinear VP needs ratio (D:L)	0.59	0.28	4.648	22	<0.001*
Linear VP needs ratio (D:L)	0.62	0.28			

Note: D:L = depth divided by length measurement.

*α = 0.05.

## DISCUSSION

Abnormalities of the velar morphology are often associated with speech and resonance disorders and, when managing children with palatal anomalies, accurate assessment of the velopharyngeal mechanism is an important component of the diagnosis and planning for surgical management. Velar length, relative to other pharyngeal dimensions, plays a role in preoperative assessment recommendations. Therefore, an MRI-based measurement technique was assessed for differing measures of velar length (Fig. 1).

Results of this study suggest that differences are present between measurements of linear and curvilinear velar length. Across all measurements, curvilinear measures were noted to be increased compared with linear measures, specifically for velar length at rest. Effective velar length did not demonstrate significant differences between measurement techniques. This is likely related to the fact that, for the majority of cases within this sample, little to no substantial curvature was present between the PMP and levator veli palatini muscle (LVP) insertion. Given the biomechanics of velar stretch in a linear (horizontal or oblique coronal) direction and its impact on the effective velar length, the minimal curvature observed within this anatomic region is likely consistent across anatomies, especially in the noncleft populations. Despite this, in cases where some curvature may exist between the PMP and LVP insertion point of the soft palate, use of a curvilinear measure may again provide a more accurate representation of the anatomic data for measurements of effective velar length. Furthermore, despite high reliability for both linear and curvilinear measurement technique, curvilinear measures likely capture the precise morphology of the velum.

**Fig. 3.**
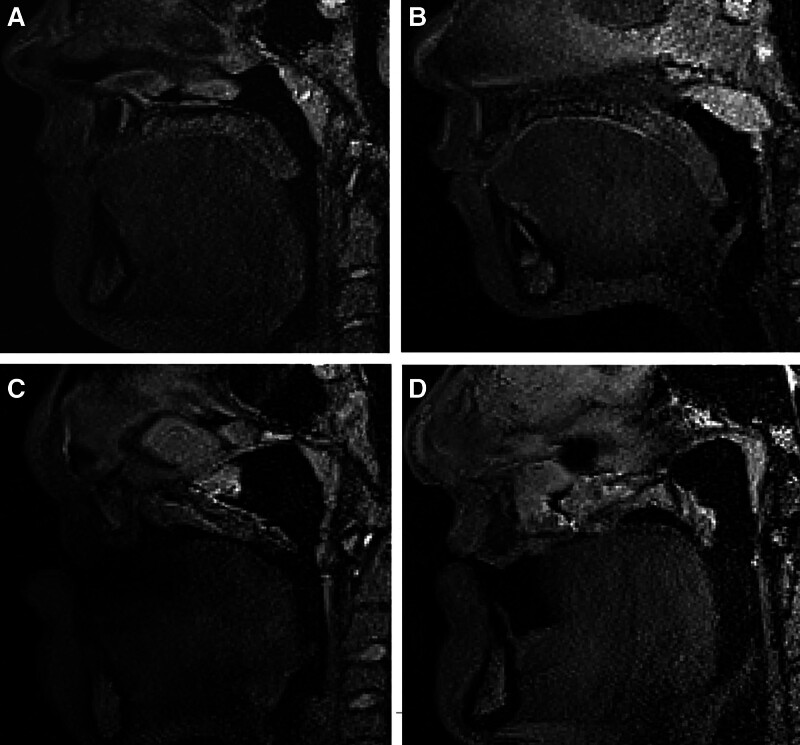
This image highlights the morphologic variability of the soft palate in individuals with varying etiologies of VPI. This underscores the need for accurate measurement of the soft palate to enhance surgical planning. A, B, Demonstration of the soft palate morphology at rest for two individuals with noncleft VPI. C, D, Demonstration of soft palate morphology during phonation in two individuals with repaired cleft palate. The curvilinear method described in this study is proposed as a solution to accurately capture the variability of the soft palate in individuals with VPI, in contrast to using a linear measurement.

Data reported in this study lend preliminary support for consistent application of curvilinear measures of velar length and have the potential to benefit future research and assessment models for predicting patient-specific outcomes. This is particularly relevant, given that recent studies have noted that small quantitative differences (<1 mm) have been reported to influence velopharyngeal function, and these differences may be correlated with the perception of hypernasality.^56,57^ For example, in patients that demonstrate velopharyngeal insufficiency, use of the curvilinear measurement provides more accurate quantitative assessment of the velar morphology and likelihood for postoperative VP function, particularly as it relates to distraction osteogenesis^30^ and obstructive sleep apnea.^58^ Furthermore, functional differences may also be observed when assessing velar length during phonation due to patient-specific anatomy postpalatoplasty, again highlighting the need to precisely measure this structure using a curvilinear measurement. Figure 3 highlights the morphologic variability of the soft palate in individuals with varying etiologies of VPI. This underscores the need for accurate measurement of the soft palate to enhance surgical planning. The curvilinear method described in this study is proposed as a solution to accurately capture the variability of the soft palate in individuals with VPI, in contrast to a linear measurement. Additional prior studies by Kotlarek et al^59^ and Kao et al^60^ further visualize morphologic variability of the soft palate in both individuals with normal anatomy and those with VPI.

A number of prior studies have additionally assessed qualitative benchmarks and velar morphology, noting significant trends toward morphological shape descriptors of the velum.^59,61,62^ Agrawal et al^61^ documented morphological differences between the velar morphology of adult male and female participants, specifically related to variations in velar shape across age groups. Craniofacial and velopharyngeal dimensions are also reported to vary based on race.^63^ Use of a curvilinear measure will likely capture these differences with greater accuracy than linear measures, particularly for patients with repaired cleft palate and those demonstrating increased variability of the velar morphology.

These measurement differences may also have scientific and clinical implications, related to calculations for the VP needs ratio. This is highly relevant, given that surgical selection for individuals with VPD, specifically pharyngoplasties, incorporates knowledge of the patient’s velar length and pharyngeal depth to identify a surgical technique that will result in improved velopharyngeal function. Underestimating velar length, if using linear measurements, may result in more radical surgical approaches being implemented, where more conservative, tailored approaches would be appropriate. Based on the findings of the present study, in cases where curvature of the velum is present, using a curvilinear measurement that tracks the precise morphology of the velum may be warranted. This is especially relevant for assessment of the soft palate during speech and sustained phonation, given the morphological variability observed during phonation. Further research is needed to fully assess the clinical implications of this measurement technique and findings from this study establish a framework for future research.

To further assess initial clinical significance of the study findings, differences in the calculated VP needs ratio were compared within subjects using linear and curvilinear measures while holding the pharyngeal depth measurement the same. Significant differences were noted between the VP needs ratio when calculated using linear and curvilinear measurement of velar length. The VP needs ratio was slightly improved using a curvilinear measure compared with the linear measure of velar length. However, questions remain regarding the significance of these measurement differences in surgical practice, as well as for making individual comparisons to existing datasets which provide normative reference data for velar length.

Furthermore, the VP needs ratio has been reported to both demonstrate variability across age groups^19^ and stay consistent across age groups.^10^ Reports on the utility of the VP needs ratio also vary across the literature.^13,15,17,29,64^ This inconsistency may be influenced by measurement technique, as both linear and curvilinear measures are utilized within these prior studies, and this warrants further study. Current results related to the consistency for measures of effective velar length, regardless of measurement technique, support findings from prior studies suggesting that the use of effective velar length to calculate the effective velopharyngeal needs ratio may be a more clinically relevant metric, given the stability of this anatomic region and consistency across age groups.^17,19,21^

Findings from the present study may also have implications for comparisons to previously established normative reference values reported in the literature. Given study findings, the measurement techniques used in future clinical and scientific reports should be explicitly and consistently documented. The imaging modality used should be considered as well. MRI provides an ideal imaging modality to assess normative benchmarks, quantify the velar anatomy, and identify parameters to define VP measurements.^2,3,50^ In comparison, cephalometric imaging can produce a lateral image, but the image may be impacted by overlapping anatomy and head rotation, making assessments of velar length less reliable than other imaging methods.^65^ MRI offers improved image resolution and the ability to identify the midsagittal image plane which eliminates the effect of head rotation. Even though radiographic methods are cost-effective and easy to use, MRI provides enhanced contrast and improved opportunities for detailed anatomic assessment.

### Limitations and Future Directions

Patients with different cleft types, palatoplasty history, and morphological abnormalities associated with velopharyngeal insufficiency and/or repaired cleft palate were included in the study sample. The inclusion of a heterogeneous group of velar abnormities may have increased the variability of the measurements. However, MRI allows for visualization of the anatomic area of interest and specifics of the cleft repair/timeline do not influence the ability to quantify the anatomy or assess differences in measurement technique. Further, generalization of results is likely improved through use of this heterogenous sample of children, given that the inclusion criteria for this study cohort is representative of the population of patients the craniofacial surgeon is likely to encounter in clinical practice. Given that this study was focused on measurement technique, rather than on analyses of the measurements themselves or development of norms, the sample composed of varying patient anatomies further supports the study purpose. Additionally, velar length was measured from the start of the PMP or posterior nasal spine, depending on the cleft status of the patient, given patients with repaired cleft palate do not demonstrate a posterior nasal spine as a clear anatomic starting point for measurement. Thus, in patients with repaired cleft palate, the estimation of the PMP was used as the starting point for measures of velar length. Despite this, inter-rater reliability was high. Increased variability in the SDs for both linear and curvilinear measurements was noted compared with normative reference values.^13^ This may be due to the fact that patients within the study sample presented with repaired cleft palate and notable morphologic variability. Future studies are necessary to determine the significance of differences in measurement technique and correlate these measurements to functional outcomes. Larger sample sizes stratified by cleft type would likely be beneficial to assess how differences in measurement technique during preoperative assessment influence intraoperative procedures related to primary palatoplasty and secondary pharyngoplasty.

## CONCLUSIONS

Results suggest that differences are present between measurements of linear and curvilinear velar length. These differences influence outcomes for the VP needs ratio. This may have implications for comparisons to previously reported normative reference values and for preoperative assessment for those who are anatomically at risk for VPI. MRI provides an enhanced assessment modality to quantify velar length and the anatomic variables used to define VP anatomy for clinical decision-making.

## DISCLOSURES

The authors have no financial interest to declare in relation to the content of this article. This work was supported in part by the National Center for Advancing Translational Sciences of the National Institutes of Health under Award Numbers KL2TR003016/UL1TR003015.

## ACKNOWLEDGMENTS

This study was approved by the University of Virginia Institutional Review Board (IRB-HSR#200333). The authors gratefully acknowledge research assistants in Imaging & Communication Outcomes Lab at the University of Virginia and the craniofacial team at UVA Health for their assistance in participant recruitment and data collection.
